# Silicone Oil Decreases Biofilm Formation in a Capacitance-Based Automatic Urine Measurement System

**DOI:** 10.3390/s21020445

**Published:** 2021-01-10

**Authors:** Martin Slettengren, Martin Linnros, Jan van der Linden

**Affiliations:** 1 Division of Perioperative Medicine and Intensive Care, Section of Cardiothoracic Surgery and Anesthesiology, Karolinska University Hospital, SE-171 76 Stockholm, Sweden; jan.vanderlinden@ki.se; 2 Department of Molecular Medicine and Surgery, Karolinska Institutet, SE-171 77 Stockholm, Sweden; martin.linnros@sll.se

**Keywords:** silicone oil, electric capacitance, albuminuria, hemoglobinuria, hemolysis, automatic urinometer, biofilm

## Abstract

Capacitance-based automatic urine measurement is a validated technique already implemented in clinical practice. However, albuminuria and free hemoglobinuria cause progressive biofilm buildup on the capacitance sensors of the urinometers. The aim of this experimental study is to investigate the influence of albumin and free hemoglobin on the capacitance signal of an automatic urinometer with and without the addition of silicone oil. A solution of Ringer’s acetate mixed with either albumin or free hemoglobin was run through an automatic urinometer containing either a water-soluble capsule with silicone oil or not. In total, around 500 capacitance measurements were retrieved from the albumin and free hemoglobin group, respectively. The mean increase in capacitance in the albumin 3 g/L group was 257 ± 100 pF without and 105 ± 30 pF with silicone oil, respectively, during 24 h. After ten hours of recording, differences between the two albumin groups reached statistical significance. For the free hemoglobin groups (0.01 g/L), the mean increase in capacitance was 190 ± 170 pF with silicone oil, and 324 ± 80 pF without, with a significant difference between the groups after 20 h and onwards. Coating of the capacitance measurement membrane of the automatic urinometer by albumin or free hemoglobin was significantly decreased by silicone oil, prolonging the functionality of the device.

## 1. Introduction

Most vital parameters in today’s intensive care units are recorded automatically, e.g., blood pressure, heart rate, and temperature. Urine output, an essential part of the fluid balance of the body, is, however, still most often recorded manually. This can lead to inaccurate recordings due to human error and/or delay, and also increases the staff’s workload. To mitigate these shortcomings, automatic urinometers have been developed, though based on different techniques: droplet-based [1], electromagnetic switch [2], high-precision scale [3], and capacitance [4]. Of these, the last method has proved the most promising and feasible for clinical use [5,6].

**Figure 1:**
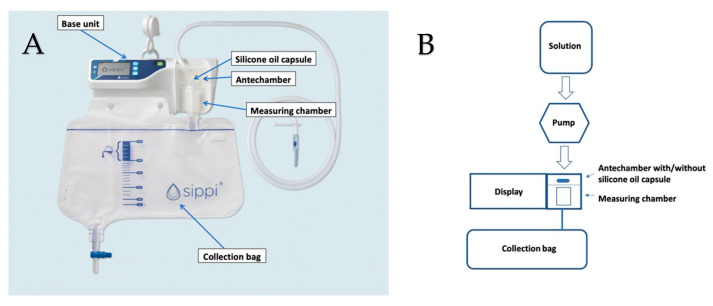
(**A**) Overview of the evaluated new automatic urinometer (Sippi^®^, Observe Medical, Gothenburg). (**B**) The experimental setup.

Sippi^®^ (Observe Medical, Gothenburg, Sweden) is an automatic urinometer based on capacitance measurements, consisting of a base unit and an attached disposable unit (Figure 1A).

The urinometer is attached to the urinary catheter from where urine flows into the antechamber. In the antechamber, the urine dissolves a capsule containing silicone oil that is transferred with the urine to the measuring chamber located just below. Change in the capacitance between two sensors is used to estimate the urine volume within the measuring chamber. After measurement, the urine departs from the chamber using a siphon technique to a collection bag. This automatic urinometer has previously been validated by our group regarding measurement accuracy in both the cardiothoracic [5] and pediatric intensive care units [6].

The capacitance of a capacitor is determined by the size and the distance between its plates, and the amount and type of the dielectric. In the automatic urinometer Sippi^®^, the dielectric consists of a column of urine in the measuring chamber. The higher the height of the urine column in the measuring chamber is, the higher the capacitance. By measuring the time it takes to charge and discharge the capacitor, a value is acquired for the capacitance, which relates to the height of the urine column in the measuring chamber. The volume of urine in the measuring chamber can then easily be calculated by the device, based on the change in capacitance. The measurement resolution is 1 mL.

Normally, biofilm is produced by local bacteria sticking to a surface. Methods to reduce biofilm-producing bacteria include surface modifications [7], antimicrobial impregnated surfaces [8], electricity [9,10], and substances to prevent the attachment of bacteria to a surface, such as cellobiose dehydrogenase/amylase [11], hydrogels [12], and silver nanoparticles [13]. Measures may also target established biofilm, e.g., signal interference between bacteria [14], antibiotic-loaded nanoparticles to enhance penetration into biofilm [15], and biofilm dispersion (e.g., Dispersin B) [16]. Lastly, the mechanical removal of biofilm is an option, but it is not easily applied in capacitance sensors. All these options, except mechanical removal, act on local bacteria that produce biofilm. In some cases—in urine, for instance—proteins such as albumin or free hemoglobin may directly form a biofilm over sensor surfaces without the involvement of bacteria. Removal of this type of biofilm is very difficult and, as far as we know, only the mechanical option is available.

A problem when using capacitance measurements to estimate urine volume is that the biofilm coating of the reading membrane will influence the capacitance signal [17], resulting in false readings, or even complete shutdown. Pilot clinical tests of the automatic urinometer Sippi^®^ in patients undergoing cardiac surgery revealed that measurements could not be recorded after 24 h in some patients who have albuminuria and/or hemoglobinuria, or a urinary tract infection. By adding a water-soluble capsule containing silicone oil to the antechamber of the device, these problems seemed to be prevented clinically, and the device could function for the recommended period of use of the disposable unit (1 week).

The aim of this study is to investigate whether albumin or free hemoglobin (fHb), common in the urine of patients following cardiac surgery, could influence the capacitance signal of the automatic urinometer, and whether this could be prevented by adding silicone oil to the measuring chamber of the device.

## 2. Materials and Methods

### 2.1. Experimental Setup

This was a prospective cohort, in vitro study investigating the effect of silicone oil on biofilm formation of albumin and fHb, each diluted in a separate solution. The studied solution was stored in a 2500 mL container with an outgoing tube, which passed through a peristaltic pump (WELCO WPX1-P3/328; WELCO Co., Ltd., Tokyo, Japan), powered by an SPS 8041 (Manson Engineering Industrials Ltd., Hong Kong, China) with a default of 4.5 volts. The outgoing tube was then connected to the ingoing tube of the Sippi^®^ device. To prevent a vacuum, air was let into the container through a small hole, created with an 18 G needle. The solution was thus run through the antechamber of the Sippi^®^, and then into its disposable collection bag (Figure 1B).

The circuit was assembled either with or without a silicone oil capsule in the antechamber. The silicone oil capsule was dissolved when the studied solution entered the antechamber, whereby the silicone oil was transferred by the solution to the measuring chamber, where it adhered to the polypropylene plastic walls [18]. Alternatively, the silicone oil capsule was removed through a hole created by a soldering iron in the front of the antechamber. Thereafter, the hole was sealed with a 3M tape designed for plastic surfaces. Every 24 h, a new solution was added to the container and the collection bag was emptied.

We used a medium-viscosity silicone oil (viscosity 350 mm^2^/s at 25 °C) (Silbione^®^, oils 70047, V350, Elkem, Oslo, Norway). This oil is a linear polydimethylsiloxane, chemically inert, heat-resistant, nontoxic, and has a low surface tension [19].

### 2.2. Albumin Solution

The first part of the study investigated the effect of an albumin solution on capacitance measurements with and without silicone oil released from a capsule. Sixty milliliters of albumin (Alburex^®^ 50 g/L, CSL Behring AB, Danderyd, Sweden) was diluted in 960 mL of Ringer’s acetate (Baxter International Inc, Deerfield, MA, USA), giving a concentration of 3 g of albumin/L. A new mixture with the same concentration, for each Sippi^®^-peristaltic pump system, was produced for every 24 h measurement. The mixture was stored in a 2500 mL container as described above. The peristaltic pump was calibrated to achieve a flow rate of approximately 42 mL/h, resulting in an estimated total protein concentration of 3 g/24 h. The albumin–Ringer’s acetate solution was conducted with two parallel groups: 20 times with silicone oil and 20 times without. Moreover, two additional experiments with a lower albumin concentration, 0.3 g/L and 1.0 g/L, respectively, were conducted.

### 2.3. Free Hemoglobin Solution

The second part of the study investigated the effect of fHb on capacitance measurement with and without silicone oil released from a capsule. fHb was acquired from blood remaining in syringes after routine arterial blood gas analysis in patients. Thirty-nine separate syringes were used. The residual blood from the syringes was centrifuged with a Sigma 1A (Axel Johnson Instruments AB, Stockholm, Sweden) at 3200 rotations per minute (RPM), for 10 min. The bottom layer, consisting of erythrocytes, was then extracted using a RAININ Pipet-lite SL1000 dropper (Rainin Instruments LLC, Oakland, CA, USA). In order to achieve lysis through osmosis, the erythrocyte concentrate was mixed with sterile water to give a volume of 10 mL. The fHb concentration was then measured with a HEMOCUE^®^ PLASMA/LOW Hb 201 + Hemoglobin spectrophotometer (HemoCue America, Brea, CA, USA). The 10 mL of fHb mixture was added to 990 mL of Ringer’s acetate and stored in 2500 mL containers as described above. Every 24 h, a new mixture, for each Sippi^®^-peristaltic pump system, was produced and used in the same way as before, with continuous capacitance measurements for 24 h. The fHb solution experiments were conducted 20 times with silicone oil and 20 times without.

### 2.4. Extraction of Data from the Automatic Urinometer

Measurements of capacitance in the measuring chamber were conducted 60 times/h, i.e., 1440 times/24 h, and data were stored on a removable micro-SD memory card inside the device. Analysis of the data was carried out after each 24 h run. In order to reach the micro-SD memory card, the batteries of the Sippi^®^ base unit were removed with a SANDVIK 7890 nippers (SNA Europe, Enköping, Sweden). The card was then inserted in a micro-SD card reader and the data were transferred to an Excel file. Every 24 h, a new disposable set was used, except for the 2500 mL container, which was reused after cleaning in a GETINGE 600 series industrial washer (Getinge Group, Gothenburg, Sweden). Before new tubes and disposable sets were connected, the hardware unit was reset, and the WELCO pumps and the SPS 8041 power unit were calibrated so that all used pumps operated at the same speed at 4.5 volts. The containers were refilled with new solution, and the pumps were restarted. Careful initial monitoring of the circuit ensured that it worked as expected and that the fluid dissolved the silicone oil capsule correctly.

### 2.5. Analysis of Capacitance Data

The Sippi^®^ registers capacitance twice every second. Capacitance is the ability to store electrical charge and is affected by the height of urine in the measuring chamber, which can then be converted by the device to a volume. A mean of the two measurements is logged to the micro-SD card of the device once every minute. From these raw data (1440 measurements/day), the lowest value from every 60 min period was extracted and stored in an Excel file, resulting in 24 measurements from every pump system per day. Twenty runs with two parallel systems (one with and one without a silicon capsule) yielded a total of 480 measurements for each group (24 × 20). Each stored capacitance value was the lowest starting point of every hourly capacitance measurement and represented, when compared with the initial starting point, the increase in capacitance due to biofilm coating of the inner surface of the measuring chamber by albumin or fHb. When the baseline value goes up, it indicates the growth of biofilm coating. Eventually, it would reach a critical point at which the measurement of urine is no longer possible.

### 2.6. Statistical Analysis

Mean and standard deviation (SD) were used for descriptive purposes. Group differences were assessed using the independent samples’ *t*-test, as the data were normally distributed. A *p*-value of less than 0.05 was considered significant. All *p*-values were two-sided. Statistical analysis was performed using the IBM SPSS Statistics for Macintosh, version 22.0 (IBM Corp., Armonk, NY, USA).

### 2.7. Ethical Considerations

Ethical permission was obtained for this study from the regional ethical review board in Stockholm (approval no. 2015-66632 and 2015/2351-32).

## 3. Results

### 3.1. Effect on Capacitance Measurement by Albumin

In total, 477 measurements without, and 472 measurements with silicone oil, respectively, were analyzed. During the 24th h, 29 measurements were excluded as readings were too few to extract a reliable average. Moreover, in a few cases, the liquid container was emptied and the pump ran dry before the end of the 24 h measurement period. The maximum capacitance value was 790 picofarads (pF) in the group without, and 633 pF in the group with silicone oil, respectively. The mean increase in capacitance was 257 ± 100 pF in the group without, and 105 ± 30 pF in the group with silicone oil, respectively. After ten hours of recording, the difference between the groups reached statistical significance (*p* = 0.011, see Supplement Table S1).

**Figure 2:**
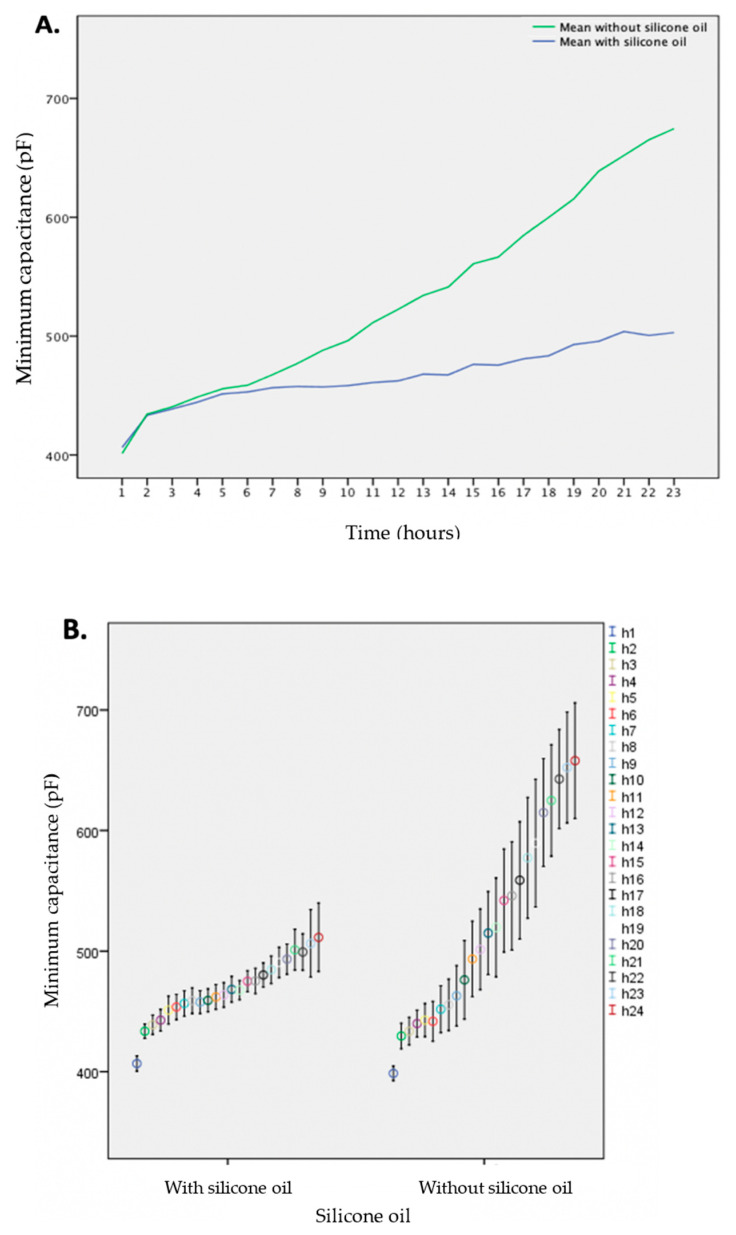
(**A**) Mean capacitance measurements caused by an albumin solution during 23 h of coating for devices without (green) and with (blue) the addition of silicone oil. (**B**) Median, maximum, and minimum capacitance values caused by an albumin solution for each hour with and without the addition of silicone oil.

The buildup of albumin coating over time is summarized in Figure 2, shown as the mean of the minimum capacitance, with and without silicone oil. The additional experiments, with 0.3 g/L and 1.0 g/L albumin solution with 379 and 190 measurements, respectively, did not show significant differences in capacitance with and without silicone oil during the 23 h time frame (data not shown).

### 3.2. Effect on Capacitance Measurements by Free Hemoglobin

In total, 484 measurements without, and 414 with silicone oil, were compared. The mean concentration of fHb in the prepared mixtures was 0.0141 g/L, ranging from 0.0056 g/L to 0.0173 g/L. The mean concentration in the group with silicone oil was 0.0113 g/L, and 0.0125 g/L in the group without. The mean increase in capacitance was 190 ± 170 pF with silicone oil, and 324 ± 80 pF without. A significant difference between the groups was seen after 20 h and onwards (*p* = 0.031, see Supplemental Table S2).

**Figure 3:**
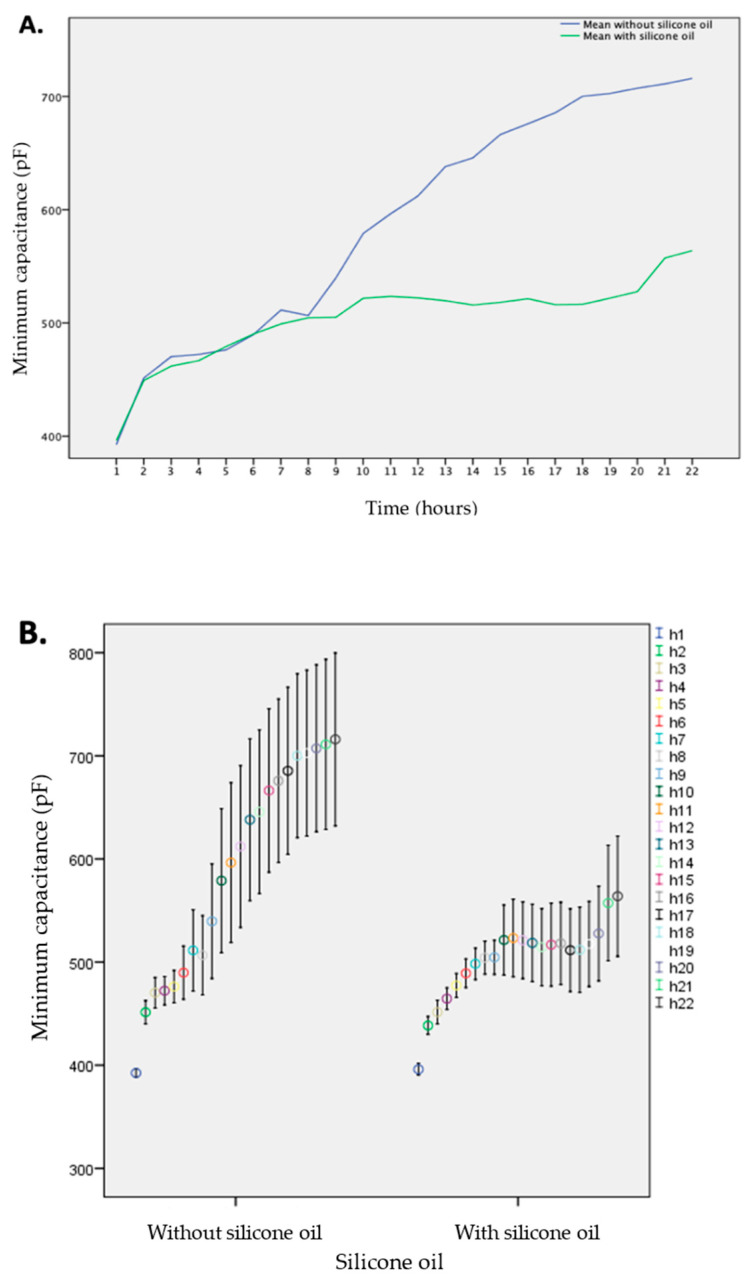
(**A**) Mean capacitance measurements caused by a free hemoglobin solution during 22 h of coating for devices without (blue) and with (green) the addition of silicone oil. (**B**) Median, maximum, and minimum capacitance values caused by a free hemoglobin solution for each hour with and without the addition of silicone oil.

The last two hours of measurements were excluded from the analysis as the readings during the 23rd and 24th h did not result in a sufficient number of readings to test for differences. Figure 3 depicts the mean increase in capacitance due to the buildup of fHb coating over time, in the group with and without silicone oil, respectively.

## 4. Discussion

Silicone oil significantly reduces the capacitance increase caused by the coating of albumin or fHb solutions on the measuring membrane of the automatic urinometer, thereby prolonging its functionality. Capacitance-based measurement is a validated technique to continuously and automatically estimate urine volume output [4,5,6]. Clinically, however, we found the technique susceptible to biofilm coating by fHb and albumin, in the urine of several patients undergoing cardiac surgery. Therefore, we investigated a new version of the automatic urinometer Sippi^®^ device that contained silicone oil in a water-soluble capsule in its antechamber.

Normally, healthy kidneys only filter very small amounts of protein into the urine, as almost all protein molecules are too large to be filtered through glomeruli. Proteinuria may be due to diseases of the glomeruli, e.g., glomerulonephritis or diabetes mellitus, urinary infection, congestive heart failure, surgery, or genetic differences in the glomerular endothelial function [20]. It is a known independent risk factor for acute renal injury [21]. In patients undergoing cardiac surgery, both preoperative [22] and postoperative [23] albuminuria can predict which patients have an increased risk of developing AKI during their hospital stay. In a large clinical study involving 1200 patients undergoing coronary surgery [23], 80% of patients had early postoperative albumin levels in urine of 0.05 g/L or lower. Thus, our studied albumin solution of 3 g/L had a significantly higher concentration than the concentration of albumin in urine found postoperatively in at least 80% of patients undergoing conventional cardiac surgery, implying that the beneficial effect of silicone oil should apply to the vast majority of conventional postoperative cardiac surgery patients.

Furthermore, early postoperative albuminuria improved the prediction of acute kidney injury to the greatest degree (clinical model area under the curve, 0.75; 0.81 with albuminuria) [23].

Hemolysis occurs during extended operations on cardiopulmonary bypass (CPB) or extracorporeal membrane oxygenation (ECMO). This may be due to mechanical forces, e.g., shear stress, hypothermia, turbulent flow, excessive pump speed, cavitation, or decreased oncotic pressure and clot formation, resulting in complete lysis or variable degrees of damage to red blood cells [24,25,26]. Excess fHb in blood is filtered by the kidneys, which excrete it into the urine, giving it a dark red color. Hemoglobinuria may lead to acute tubular necrosis, acute renal failure, and need for dialysis. In a study by Heijmans et al. [27], patients undergoing extended periods of CPB (valve + coronary surgery) had higher levels of plasma fHb than those undergoing shorter periods (coronary surgery) during CPB and the first postoperative hours. In contrast, patients undergoing off-pump coronary surgery did not have increased levels of fHb in plasma. Patients with AKI (13.4%) exhibited significantly higher fHb already during surgery, compared with patients without AKI.

Both the solution containing albumin (3 g/L), and that containing fHb, significantly increased the capacitance measurement in the automatic urinometer, perhaps by changing the properties of the dielectric between the plates that determine the measured capacitance values, and thus the estimated urine volume. One theory is that the electrical quantities change depending on the excitation quantities, which in this study corresponds to the electrical capacity dependence on the albumin concentration and on the free hemoglobin concentration in the urine [28]. Importantly, the urinometer will initially, despite the increased biofilm coating of the measurement membrane, quantify the urine volume correctly until a certain cut-off value is reached, whereupon the urinometer will shut down prematurely. The rises in capacitance were clearly and significantly lower when the urinometers contained silicone oil during the 23 h study period, partially preventing biofilm formation in the measuring chamber.

The siphon cassette of the studied automatic urinometer, consisting of the antechamber and the measuring chamber, is made up of polypropylene plastic, which is inherently hydrophobic and oleophilic [29]. Due to the nonpolar and hydrophobic properties attributed to polypropylene, silicone oil will accumulate on the surface of polypropylene. Based on the chemical structure and an initial pilot study using Raman microscopy (unpublished data), some amount of the hydrophobic oil will attach to the polypropylene plastic surface, in the form of minute droplets, and in doing so, exert the long-lasting, clinically observed effect, despite surfaces being intermittently exposed to air between each emptying of the measuring chamber.

The clinical consequence of the addition of silicone oil is that urine volume measurements with this capacitance-based automatic urinometer can be reliable for longer periods, before the disposable part with the measuring chamber needs to be replaced in patients who have hemoglobinuria and/or albuminuria, e.g., early postoperatively after cardiac surgery with CPB, and in patients with diabetes mellitus and renal dysfunction. Indeed, our initial clinical observation of failed measurements early after cardiac surgery was not seen in any patients after the inclusion of the water-soluble capsule containing silicone oil, whereupon the urinometer was still functioning 7 days later, which is the recommended period of use of the disposable unit. To our knowledge, this is the first study indicating at least partial protection of an albumin and fHb biofilm, respectively, on plastic surfaces with use of silicone oil.

This study has a few limitations. First, we used Ringer’s acetate instead of real urine. We could have used more complicated formulas for artificial urine [30], but we regarded Ringer’s acetate an appropriate, cheap, and easily available solution to mix with albumin and fHb. In contrast, it would have been complicated and costly to produce large volumes of sterile artificial urine. Another alternative would have been to use human urine. However, that would also have been complicated considering the large volume needed (approximately 80 L), the great interindividual variation in electrolyte composition and pH, as well as keeping these large volumes sterile. Moreover, other studies have used similar solutions as a replacement for human urine, e.g., Rasmussen et al., investigated ascending infections using 0.9% saline [31]. Second, if our experiments had lasted more than 24 h, we could have seen significant effects with the 0.3 and 1.0 g/L albumin solution, respectively. Third, the concentration of fHb differed slightly between each run. However, the mean difference in concentration was very similar between both groups and should consequently only marginally have influenced the results. Interestingly, the fHb concentration in urine in patients with discolored urine in the first hours after cardiac surgery with CPB usually vary between 0.1 and 5 g/L in our cardiothoracic intensive care unit, after which it usually drops. Thus, in our study we used an fHb concentration that was approximately 10% of the real values at most. On the other hand, since the introduction of the built-in silicon capsule in the disposable part of the Sippi^®^ device, we have not experienced malfunctioning measurements in our clinical routine. Fourth, the study was not randomized or blinded, but as data measurements were automatic this should not have influenced the results.

In summary, coating by albumin or fHb of the capacitance measurement membrane of an automatic urinometer can be significantly decreased by integrating a dissolvable capsule of silicone oil in the antechamber of the device, and in doing so, prolong its functionality.

## Data Availability

The data presented in this study are available in Supplemental Tables S1 and S2.

## Supplementary Materials

The following are available online at https://www.mdpi.com/1424-8220/21/2/445/s1, Table S1: Capacitance parameters and change over 23 h with albumin solution (3 g/L), Table S2: Capacitance parameters and change over 22 h with free hemoglobin solution (0.01 g/L).
