# Supplementary material for: Silicone Oil Decreases Biofilm Formation in a Capacitance-Based Automatic Urine Measurement System
Source: Sensors (Basel). 2021 Jan 10;21(2):445. doi: 10.3390/s21020445 (PMC7826702; doi:10.3390/s21020445)
Supplement: Supplementary file 1 [file sensors-21-00445-s001.zip › Supplemental tables/Table S2.pdf]

Table S2. Capacitance parameters and change over 22 hours with free hemoglobin solution (0.01 g/L).

	**With silicone oil (n=414)**					**Without silicone oil (n=484)**					
**Hour**	Min cap	Max cap	Median	Mean min cap	SD	Min cap	Max cap	Median	Mean min cap	SD	P-value (mean min cap)
**1**	379	414	396	396	11	378	411	393	393	10	0.09
**2**	406	641	449	449	49	404	496	451	451	26	0.266
**3**	398	650	462	462	51	416	563	470	470	33	0.272
**4**	427	530	467	467	22	417	534	472	472	31	0.542
**5**	432	539	479	479	24	417	561	476	476	35	0.649
**6**	432	561	490	490	27	408	691	490	490	58	0.642
**7**	433	575	499	499	30	414	773	511	511	89	0.652
**8**	434	591	505	505	31	414	798	507	507	87	0.327
**9**	435	601	505	505	32	411	843	540	540	125	0.724
**10**	430	752	522	522	66	415	881	579	579	158	0.622
**11**	429	779	523	523	74	404	880	596	596	175	0.922
**12**	431	765	522	522	73	404	874	612	612	177	0.518
**13**	433	767	520	520	74	410	873	638	638	177	0.257
**14**	431	761	516	516	73	403	862	646	646	179	0.232
**15**	438	783	518	518	79	399	866	666	666	178	0.164
**16**	451	777	521	521	79	422	873	676	676	179	0.176
**17**	456	767	516	516	81	421	877	686	686	183	0.091
**18**	454	776	516	516	83	402	880	700	700	179	0.058
**19**	448	784	522	522	83	406	873	703	703	181	0.06
**20**	433	775	528	528	93	420	878	707	707	183	0.031
**21**	428	818	557	557	113	409	897	711	711	186	0.037
**22**	428	824	564	564	117	412	907	716	716	189	0.028
