# Supplementary material for: Silicone Oil Decreases Biofilm Formation in a Capacitance-Based Automatic Urine Measurement System
Source: Sensors (Basel). 2021 Jan 10;21(2):445. doi: 10.3390/s21020445 (PMC7826702; doi:10.3390/s21020445)
Supplement: Supplementary file 1 [file sensors-21-00445-s001.zip › Supplemental tables/Table S1.pdf]

Table S1. Capacitance parameters and change over 23 hours with albumin solution (3 g/L).

	**With silicone oil (n=472)**					**Without silicone oil (n=477)**					
**Hour**	Min cap	Max cap	Median	Mean min cap	SD	Min cap	Max cap	Median	Mean min cap	SD	P-value (mean min cap)
**1**	381	433	405	406	12	382	415	406	401	10	0.176
**2**	413	458	433	433	11	406	464	438	434	17	0.56
**3**	417	471	439	439	15	407	482	437	441	18	0.839
**4**	417	479	447	444	17	418	488	443	449	19	0.756
**5**	406	510	454	451	21	426	506	446	456	26	0.914
**6**	422	497	452	453	19	416	554	450	459	36	0.903
**7**	426	506	457	457	19	420	566	457	468	39	0.675
**8**	426	508	455	458	19	418	583	460	477	45	0.386
**9**	423	499	454	457	17	423	590	479	488	48	0.062
**10**	425	500	458	458	18	419	614	485	496	51	0.011
**11**	419	507	460	461	19	436	614	503	511	52	<0.001
**12**	425	508	461	462	19	437	627	522	523	57	<0.001
**13**	428	505	473	468	20	454	649	524	534	58	<0.001
**14**	436	487	469	467	15	467	670	524	541	66	<0.001
**15**	441	500	480	476	17	456	716	560	561	71	<0.001
**16**	430	503	478	476	19	447	727	545	567	76	<0.001
**17**	440	513	485	481	19	454	760	572	585	81	<0.001
**18**	441	520	488	483	22	452	754	596	600	79	<0.001
**19**	440	544	490	493	24	452	759	645	615	81	<0.001
**20**	437	535	497	496	24	479	729	659	639	65	<0.001
**21**	439	554	502	504	32	470	788	659	652	71	<0.001
**22**	440	556	502	501	27	494	790	670	665	63	<0.001
**23**	400	660	503	503	56	472	785	677	674	67	<0.001
